# Genome reduction in marine ammonia‐oxidizing archaea driven by elevated temperature

**DOI:** 10.1002/mlf2.70099

**Published:** 2026-09-21

**Authors:** Minglei Ren, Jianjun Wang

**Affiliations:** ^1^ State Key Laboratory of Lake and Watershed Science for Water Security, Nanjing Institute of Geography and Limnology Chinese Academy of Sciences Nanjing China

## Abstract

Marine ammonia‐oxidizing archaea (AOA) have evolved into diverse lineages and ecological niches. However, less is known about the impact of environmental factors on their genomic evolution. By integrating 281 AOA metagenome‐assembled genomes (MAGs) around global oceans, we revealed that AOA genome size decreased with elevated temperature across the two predominant genera *Nitrosopumilus* and *Nitrosopelagicus*. This was supported by their contrasting temperature preferences, with the large‐genome *Nitrosopumilus* commonly occupying cold waters and the genome‐reduced *Nitrosopelagicus* preferring warm waters. We further found a warm‐like subclade diverging from the cold‐adapted *Nitrosopumilus* and a cold‐like subclade from the warm‐adapted *Nitrosopelagicus*. These divergences were accompanied by the enrichment of functional genes associated with adaptation to temperature fluctuation. Our results highlight the role of temperature in shaping genomic reduction and phylogenetic divergence in marine AOA, with implications for nitrogen cycling under ocean warming.

Ammonia‐oxidizing archaea (AOA) of the *Nitrososphaeria* class (classified as *Nitrososphaeria* in the GTDB system, formerly the *Thaumarchaeota* phylum) are prevalent in marine ecosystems, comprising up to 39% of microbial plankton in the ocean^1^. As key members mediating the first step of the nitrification processes, AOA play an important role in the marine nitrogen cycle. Benefiting from extensive cultivation efforts, a handful of archaeal isolates are well characterized among two distinct lineages of marine AOA, including *Nitrosopumilus* ^2^ and *Nitrosopelagicus* ^3^. These diverse marine AOA show substantial variation in genome size, such as 1.23 Mbp for *Nitrosopelagicus brevis* CN25 and 2.17 Mbp for *Nitrosopumilus ureiphilus* PS0. In addition, the advances in cultivation‐independent metagenomics have revealed that marine AOA show physiological flexibility and thrive across wide gradients of water depth and temperature^4^, ^5^, ^6^. Despite these genomics‐based findings, major gaps remain in our understanding of the relative contribution of environmental factors to the diversification and evolution of marine *Thaumarchaeota*. In this study, we performed comparative genomic analyses of thaumarchaeotal metagenome‐assembled genomes (MAGs) across the global ocean to explore how environmental factors are associated with genomic evolution. Our results provide evidence for the warming‐related genomic reduction in marine AOA and their diversification toward new niches.

We found that marine AOA were dominated by two predominant genera, which show genome size variation across wide environmental ranges around the global ocean. To quantify the geographic distribution of marine AOA, the conserved ammonia monooxygenase alpha subunit gene (*amoA*) was searched against a global ocean microbial gene database^7^. Phylogenetic assignment of the retrieved *amoA* showed that marine AOA were dominated by *Nitrosopumilus* and *Nitrosopelagicus* (Figure 1A) by accounting for 81.21% and 18.75% of the relative abundance, respectively. The predominance of both genera indicated that they could well represent the marine AOA community. By further integrating the ocean microbial genome dataset and the relevant physicochemical factors of the original metagenomes^8^, we found that there were 152 and 129 MAGs belonging to the genera *Nitrosopumilus* and *Nitrosopelagicus*, respectively (Figures 1A and S1, and Table S1). Among these MAGs, the genome size varied from 0.94 to 1.54 Mbp for *Nitrosopumilus* (mean: 1.19 Mbp), whereas it ranged from 0.88 to 1.34 Mbp for *Nitrosopelagicus* (mean: 1.11 Mbp). The size of *Nitrosopumilus* genomes was significantly larger than that of genomes within *Nitrosopelagicus* (*p* < 0.001, Wilcoxon test), consistent with the estimation from pure cultures. Unexpectedly, marine AOA within two genera showed wide environmental ranges, with temperatures ranging from 1.6°C to 27.7°C, water depth from 0 to 5100 m, dissolved oxygen from anoxic to 414.9 µmol/l, and salinity from 3.0 to 40.5 psu (Table S1 and Figure S2).

**Figure 1:**
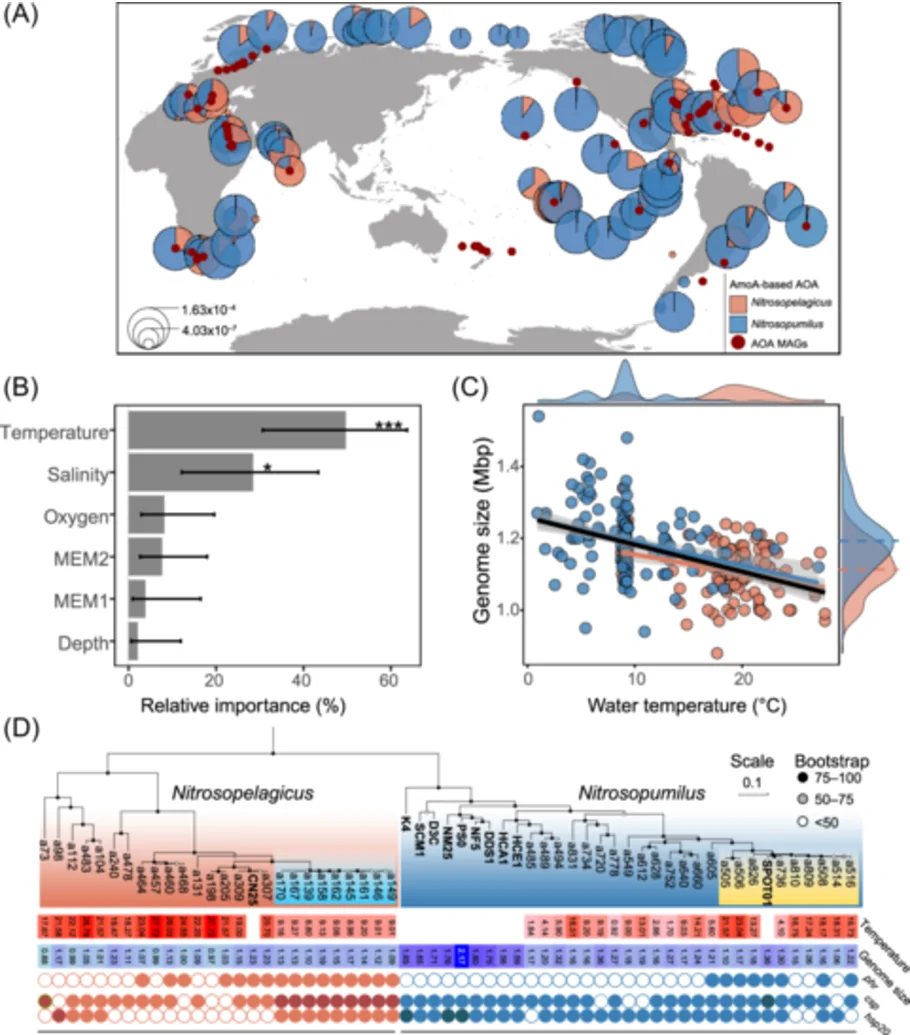
Distribution and phylogeny of marine AOA in the global ocean and their relationships with water temperature. (A) Relative abundance of the AmoA‐based marine AOA and the distribution of AOA MAGs across the global ocean. The background world map was downloaded from the government website: http://bzdt.ch.mnr.gov.cn/. The size of each circle on the map represents the relative abundance of all marine AOA identified at each sampling site, expressed as transcripts per million, with larger circles indicating higher relative abundance. (B) Relative contribution of environmental factors to the variation in marine AOA genomes. The geographical variables of MEM1 and MEM2 were the first two dimensions of the dbMEM eigenvalues, which were calculated using the locations of the MAGs. The error bar represents the bootstrap confidence intervals for relative importances using the linear model calculated by the LMG method, and the star represents the statistical significance of the relationships. *p < 0.05 and ***p < 0.001. (C) Relationship between water temperature and marine AOA genome size across both genera. The point represents the marine thaumarchaeotal genome in each sample. (D) Phylogenomic tree of marine AOA species, including 48 MAGs and 11 pure species (bold), and enrichment of functional genes. These 48 AOA MAGs were representative genomes selected from the total of 281 MAGs, with details described in the Supporting Information. The maximum likelihood tree was built based on the concatenated alignment of 72 core gene families, further rooted by two terrestrial AOA species, which were suppressed in the plot. Two diverged clades with distinct temperature preferences were highlighted with colors different from the background colors. The annotated functional genes significantly associated with water temperature were identified using generalized linear models. For each gene, the darker color indicates a multiple copy and an empty circle indicates the absence of the gene. The detailed information regarding the reference genomes is shown in Table S2, the tree file is shown in Table S3, and the genes are shown in Table S4. AOA, ammonia‐oxidizing archaea; MAG, metagenome‐assembled genome; MEM, Moran's Eigenvector Map; LMG, Lindemann, Merenda, and Gold; phr, deoxyribodipyrimidine photolyase.

We further explored the relative importance of environmental factors on the genome size variation of marine AOA (details of methods are described in the Supporting Information section). We found that water temperature explained 49.67% of the variation in genome size, followed by salinity (28.50%), dissolved oxygen (8.18%), geographical locations (11.56%), and water depth (2.09%) (Figure 1B). Consistently, the genome size of marine AOA decreased with elevated temperature across the genera of *Nitrosopumilus* and *Nitrosopelagicus* (*R* ^2^ = 0.262, *p* < 0.001, Figure 1C), and the significant relationships were also true for each genus (*Nitrosopumilus*, *R* ^2^ = 0.105, *p* < 0.001; *Nitrosopelagicus*, *R* ^2^ = 0.131, *p* < 0.001). Compared with temperature, the marine AOA genome sizes were also weakly correlated with water depth, dissolved oxygen, and salinity (Figure S2). These correlations indicate distinct roles of different environmental factors in driving genomic evolution and potential niche expansion of marine AOA. The hypothesis was further supported by the distinct growth temperature among marine AOA. For example, we found that marine AOA in warming waters also showed a higher optimal growth temperature (Figure S3), a key life history trait reflecting species' thermal adaptation.

We next explored how gene composition and functional categories varied with temperature to infer potentially adaptive strategies using pangenome analyses. In line with the genome size distribution, water temperatures were correlated with the number of orthologous families. For example, the genus *Nitrosopelagicus* showed fewer orthologous genes in the total, core, and flexible groups toward higher temperature, though the relationship was weak within *Nitrosopumilus* (Figure S4A). In addition, we further observed that the proportion of flexible genes in each genome decreased more rapidly than core genes, though flexible genes generally considered to be less conserved in microbial pangenomes. When examining functional composition, marine AOA in warming waters harbored fewer genes related to transcription regulation, cell wall/membrane biogenesis, and the metabolism of amino acids and carbohydrates (Figures S4B and S5). Specifically, more metabolic pathways in the genus *Nitrosopelagicus* were shown with decreasing patterns toward higher temperature when compared to *Nitrosopumilus*. The decreasing proportion of specific functional categories for marine AOA indicates their ecological adaptation to cold and/or warm conditions.

The phylogenomic tree revealed that both genera of marine AOA harbored the niche‐specialized clades, including a warm‐like clade within *Nitrosopumilus* and a cold‐like one within *Nitrosopelagicus*. These MAG‐represented species greatly expanded the phylogenetic diversity of marine AOA, resulting in diverse lineages distinct from the cultivated species (Figure 1D). Within *Nitrosopumilus*, a diverged subclade containing a cultivated archaeon and nine MAGs showed a significant preference for the environments with elevated water temperature (16.6 ± 5.47°C, *p* < 0.001, Wilcoxon test) when compared to other MAGs (7.49 ± 5.24°C). The warm‐like subclade had an average within‐clade ANI (average nucleotide identity) of 92.50% (86.87%–97.74%), while the average ANI of the subclade and other genomes in this genus was 81.75% (77.89%–88.53%). Within *Nitrosopelagicus*, the cultivation‐independent MAGs represented all tips, except for a type strain *N. brevis* CN25, indicating that a great majority of marine AOA species remain uncultured. In addition, the genus *Nitrosopelagicus* was a very recently diverged clade preferring cold waters (9.07 ± 0.14°C) when compared to other MAGs (22.50 ± 3.25°C). The cold‐like subclade, which consisted of nine MAGs, had an ANI of up to 99.58%, while it showed higher divergence with the strain CN25 (mean ANI of 87.35%) and the remaining MAGs (83.13%) within the genus. Moreover, these diverged clades had optimal growth temperatures differing from that of their ancestral clades (Figure S6). The difference in predicted growth temperatures was significant in the genus *Nitrosopelagicus* though weak in *Nitrosopumilus*, corroborating niche expansion of marine AOA.

We further identified the genes potentially underlying the diversification of marine AOA and their adaptation to growth at low or high temperatures using gene enrichment analyses (Figure 1D). Functional gene enrichment analyses showed that there were 151 significantly temperature‐associated functional gene families in *Nitrosopumilus* and 202 in *Nitrosopelagicus* (Table S4). Among these functional genes, we found that most of the members of the warm‐like subclade within *Nitrosopumilus* harbored a gene encoding deoxyribodipyrimidine photolyase (phr), which is responsible for the repair of DNA damage induced by UV radiation^9^. This metabolic potential likely enables members of the subclade to cope with light‐induced stress and grow well in warming surface waters when compared to deeper cold environments. Compared to the warm‐adapted *Nitrosopelagicus*, the recently diverged cold‐like subclade in the genus enriched more chaperone‐encoding genes involved in low‐temperature adaptation, including the cold shock protein Csp and small heat shock protein Hsp20 families (Figure 1D). The Csp protein has been shown to be involved in adaptation to cold temperatures in psychrophilic and mesophilic archaea^10^, whereas the Hsp proteins are experimentally demonstrated to improve microbial tolerance to a wide range of physical stressors, including low temperatures^11^.

Microbial genomic evolution has been shaped by environmental factors, which is exemplified by a wide range of studies. For example, marine prokaryotic genome size is associated with water depth for benthic and pelagic communities^12^, and the larger genome size, likely driven by nutrient availability, is also observed among diverse microbial clades across depth profiles in the ocean^13^. In this study, we found that the genome size of marine AOA was primarily driven by temperature, with much weaker associations with water depth and other factors. The stronger effect of temperature is parsimoniously explained by the effect of temperature on the rate of cellular metabolic processes^14^ and mutation rates^15^, which are associated with genome size reduction in prokaryotes^16^. Temperature also strongly influences the distribution and composition of marine AOA community in the ocean^5^. Consistently, a previous study demonstrates that temperature induces genome size changes in marine microbes at rates more than 10 times higher than those induced by water depth^17^.

The role of temperature in shaping AOA divergence has been largely overlooked, despite long‐standing observations that marine AOA form depth‐related phylogenetic groups^4^. In this study, we show that marine AOA are adapted to distinct thermal niches. This is supported by two observations, namely, that the predicted optimal growth temperatures of marine AOA correlate strongly with their ambient temperatures (Figure S3), and the cold‐ and warm‐adapted subclades are well separated (Figure 1D). AOA with smaller genomes tend to inhabit the warmer, light‐rich pelagic zone. The mutagenic effects of UV light‐induced DNA damage in the surface layer may be alleviated by the presence of photolyase in both genera. Previous studies have identified two distinct photolyase‐related gene families in marine AOA genomes, including the genus *Nitrosopelagicus* ^18^. For *Nitrosopumilus*, one family (the FAD binding domain of DNA photolyase, arCOG02840) is largely present in the isolates as shown previously^19^, while the other one (deoxyribodipyrimidine photolyase‐related protein, COG3046) was enriched in the warm‐like subclade, including one isolate (SPOT1) and the other MAGs. The enriched pattern indicates that the photolyases are likely involved in the adaptation of the warm‐like *Nitrosopumilus* to the surface layer. It seems that the genes play a minor role in the adaptation of the *Nitrosopelagicus*, being scattering across the cold‐adapted clade and other MAGs of the warm‐adapted clades within the genus. The enrichment of chaperone‐related genes, including the cold shock protein Csp and small heat shock protein Hsp20, provides a possible explanation for the low‐temperature adaptation of the cold‐adapted clade of the genus. Interestingly, the Csp protein was previously proposed to have been acquired by the most recent ancestor of *Nitrosopumilales* from bacteria to adapt to lower temperatures in marine environments^20^.

In summary, our study provides novel insights into the role of water temperature in shaping genome size and divergence of AOA through phylogenomic analyses of 281 MAGs across the global ocean. We revealed that genome reduction in marine AOA was mainly driven by water temperature, showing a small genome size toward elevated temperature across predominant genera. The divergences within both genera are accompanied by temperature‐mediated niche expansion, which is likely promoted by the enrichment of key genes involved in their adaptation to fluctuating temperatures. Our results highlight that temperature significantly affects the genome content variability and divergence of marine AOA, key players in nitrogen cycling. Together with the direct influence of temperature on the growth and activity of marine nitrifiers, the increasing ocean temperatures under climate change would cause profound impacts on global nitrogen cycling.

## ETHICS STATEMENT

This study did not involve any human participants or animal subjects.

## Supporting information

Supporting Information.

Supporting Information.

## Data Availability

All genomes are available from public databases, and their accession numbers and associated environmental information are provided in the Table S1.
