# Supplementary material for: Genome reduction in marine ammonia‐oxidizing archaea driven by elevated temperature
Source: mLife. 2026 Sep 21;5(5):675–9. doi: 10.1002/mlf2.70099 (PMC13591554; doi:10.1002/mlf2.70099)
Supplement: Supplementary file 1 [file MLF2-5-675-s002.docx]

Genome reduction in marine ammonia-oxidizing archaea driven by elevated temperature

Minglei Ren^1, 2^, Jianjun Wang^1, 2, #^

^1^ State Key Laboratory of Lake and Watershed Science for Water Security, Nanjing Institute of Geography and Limnology, Chinese Academy of Sciences, Nanjing 211135, China.

^#^ Correspondence: jjwang@niglas.ac.cn

**Supplementary Materials and Methods**

**Estimating the global distribution of marine AOA**

Before diving into genomic evolution, we firstly explore the distribution of marine AOA based on the conserved marker genes. The relative abundance and geological location of marine AOA around the global ocean were evaluated using the web service of the Ocean Gene Atlas database ^1^. The online service enables to explore the global biogeography of marine plankton through identifying the marine environmental homologues based on user’s query and providing the relative abundance normalized by sampling sequencing depth. Marine AOA was represented by a conserved marker gene, the ammonia monooxygenase alpha subunit (*amoA*). We firstly searched the marker gene represented by PF12942 (Archaeal_AmoA) in the Pfam database, against the TARA ocean microbiome reference with HMMER ^2^ using the default setting in the web server, then downloaded the results, including the hit *amoA* homologues, their abundance and the contextual geological information across global ocean samples. The relative abundance was computed in RPKM (reads per kilo base covered per million of mapped reads. We next reconstructed a phylogenetic tree consisting of the *amoA* genes from the environmental microbiomes and the reference AOA genomes, to determine the taxonomy of the hit *amoA*. The details for the phylogenetic analysis were shown below section.

**Genome downloading and cleaning**

We retrieved 959 metagenomic-assembled genomes (MAGs) belonging to the phylum Thaumarchaeota (now classified as the Nitrososphaeria class of the Thermoproteota phylum in GTDB taxonomy) from the oceanDNA MAG database ^3^. This database contains 52,325 draft-quality genomes from 2,057 metagenomes around the global ocean, as well as a wide range of physicochemical factors associated with environmental samples. The genome quality, including genome completeness and contamination, were estimated by CheckM v1.2.4 ^4^. Initially, we filtered the genome dataset with genome completeness ≥ 80% and contamination ≤ 5%, resulting in 402 MAGs with 329 assigned to the Nitrosopumilaceae family, and the remaining 73 distributed in the lineages either without culture representatives (the family UBA213 and UBA141 assigned in the GTDB taxonomy classification) or the non-AOA lineages of the phylum (the family UBA57). Our study here focused on the genomes of the family Nitrosopumilaceae, considering the numerically dominance of the family in the dataset and the increasing number of cultivated strains within the family ^5-8^. Finally, we obtained 281 high-quality MAGs from 230 metagenomic samples, with 129 and 152 belonging to the genus *Nitrosopumilus* and *Nitrosopelagicus* within the family Nitrosopumilaceae, respectively, after discarding the genome without environmental parameter available. Furthermore, we downloaded 13 reference genomes of enriched and pure thaumarchaeotal strain from the GenBank database, including 10 from the genus *Nitrosopumilus*, one from the genus *Nitrosopelagicus* of the family Nitrosopumilaceae, and two from the genus *Nitrososphaera*. These reference genomes of the family Nitrosopumilaceae were included due to their high completeness (98.50% - 100%), whereas the last two *Nitrososphaera* AOA strains isolated from soils were used as the outgroup in the downstream phylogenetic analyses. The accession numbers for all AOA MAGs and relevant statistics were shown in the Supplementary Table S1, and the information for the reference genomes in Table S2.

**Estimating the importance of environmental factors to genome size variation**

To investigate the effect of environmental factors on genome content, we consider four physicochemical and two geographical factors in the study. The four environmental factors, namely, water temperature, water depth, dissolved oxygen, and salinity, were retrieved from the oceanDNA MAG database mentioned above. The spatial variables for the MAGs were represented by the first two dimensions of the distance-based Moran's eigenvector maps (dbMEM), which were calculated based on the geographic distance matrix by the ‘dbmem’ function implemented in the adespatial package v0.3.21 ^9^. The variables represent a spectral decomposition of the spatial relationships among the samples where these MAGs were reconstructed. Before calculating the relationships, the genome size of the AOA MAGs was calibrated with genomic completeness and contamination using *G`* = *G* /(( 1 + *T* ) * *C*), a formula described elsewhere ^10^. In the formula, *G* and *G`* are the estimated and corrected genome size of a MAG, *T* and *C* are the estimated genomic completeness (0-1) and contamination (0-1).

To tell which variables was more important to explain the variation in marine AOA genome size, we calculated the relative contribution of each variable to the R square with the consideration of the sequence of variables using the LMG method ^11^. We used both spatial variables and environmental variables as explanatory variables, and genome size as the response variable. Firstly, we built a linear regression model fitting the relationships between marine AOA genome size and all explanatory variables. We next estimated the relative importance of each of explanatory variables for the linear model and their bootstrap confidence intervals using the LMG method, which was implemented by the ‘boot.relimp’ function in relaimpo package v2.2.6 ^12^.

**Ortholog identification and pangenome analysis**

For pangenome analyses, homologous gene families among 281 marine AOA MAGs and AOA reference genomes were identified using OrthoFinder v2.5.4 ^13^, with the DIAMOND v2.1.10 serving as the sequence search engine ^14^. The bioinformatic programs here and below were called with the default parameters, except the detailed explanation. For each genus, all homologs were classified into three groups, with the homologs found in at least 90% species assigned to the ‘core’ group, less than 1% species (n = 2) to the ‘singleton’ group, the remaining as the ‘flexible’ group (present in at least 1% but no more than 90% species). Noted that a relatively relaxed definition of the core homologs, i.e. 90%, rather than 100%, in order to avoid the bias of metagenomic fragmentation and uneven genomic completeness on pangenome analyses ^15^.

**Genome annotation and genomic features**

The protein-coding sequence (CDS) for each archaeal genome was predicted using Prodigal v2.6.3 ^16^ with the ‘-single’ parameter, then functionally annotated with eggNOG_mapper ^17^ against EggNOG database v5.0 ^18^. We next predicted the optimal growth temperature for marine AOA MAGs using tome, which is based on the proteome-wide amino acid composition ^19^. The pair-wise genomic average nucleotide identity (ANI) values were calculated using fastANI v1.33 ^20^. The 40 species-level genomes were selected from all 281 MAGs based on the threshold of 97% genome-wide ANI. For the clusters with multiple genomes, the one with the highest completeness and lowest contamination was selected as the representative.

**The construction of phylogenomic trees**

Two sets of environmental genomes were used to construct the phylogenetic trees. Specifically, the first consisting of 281 redundant MAGs, and the second containing 48 representative MAGs. The latter set comprised 40 species-level genomes and eight *Nitrosopelagicus*-related genomes with genomic ANI greater than 97% but exclusively inhabiting low temperature environments (Table S1). The first resulting tree showed the patterns of all redundant MAGs whereas the other tree showed phylogenetic relationships of the species-level AOA MAGs and reference genomes.

The construction of the phylogenomic trees were performed using a concatenated alignment of the conserved orthologous genes as described previously ^21^. Briefly, the orthologous genes were aligned at the inferred amino acid sequence level using MAFFT v7.490 ^22^, trimmed using TrimAl v1.4.1 ^23^ with the ‘-automated1’ option. For the first genomic dataset, 85 single-copy orthologs present in at least five marine AOA strain and two AOA MAGs were identified and concatenated into 11,158 sites. For the second dataset, 71 single-copy orthologs present in all 13 reference AOA strains were identified and concatenated into 5484 sites. For the first genome dataset, an approximately maximum-likelihood tree was built by FastTree2 v2.1.11 with the default setting ^24^, whereas a maximum-likelihood tree for the second dataset was built by IQTree2 v2.1.2 ^25^, with the substitution model determined by ModelFinder ^26^. The analysis employed an edge-linked partition model and included 1,000 replicates via an ultrafast bootstrap approximation. The resulting phylogenetic tree files were shown in Newick format in the Supplementary Table S3.

**The enrichment of functional genes along the temperature gradient**

We next identified functional genes that were potentially involved in the adaptation to temperature changes using functional gene enrichment analyses approach. We determined the functional genes that were significantly associated with water temperature by modeling their presence in each marine AOA MAG as a function of temperature with generalized linear models (GLM) with a binomial distribution, as described previously ^27^. Briefly, in a single GLM model for the ortholog, its presence/absence across the genomes was the response variable and temperature as the independent variable using the logit as a link function implemented by the ‘glm’ function in the ‘stats’ package. For each genus, only the orthologs that were present in greater than 5% and less than 95% MAGs, were included in the model fitting. The *P* values of the models were then corrected using the Benjamini and Hochberg method to control false discovery rate ^28^. Only the genes with the *P* values less than 0.05 were further manually inspected to determine the candidate of genes potentially involved in temperature-associated adaptation. The detailed statistics regarding the temperature-associated functional genes in marine AOA were shown in Table S4.

**Supplementary text**

**Measures to minimize potential effect of genome completeness on genome size**

The estimation of genome size may be affected by assembly quality when no or few enrichments or isolates are available. Three measurements in the study were performed to minimize the effect of genome completeness on our result. Firstly, the high-quality MAGs (genome completeness ≥ 80% and contamination ≤ 5%) combined with AOA strains with full genomes were included in the functional analyses. Secondly, genome size of target MAGs was corrected by genome completeness and contamination. Finally, the observed genome size pattern was further supplemented by the pangenome analyses. Specifically, the trend of core and flexible genome contents along the temperature gradient further supported the genome size pattern (Fig. 2a). Anyway, the relationship between marine AOA and water temperature needs to be further validated by pure culture or single cell genomics combined with environmental physiochemical factors.

**Supplementary Figures**

**Figure S1.** The distribution and phylogeny of AOA MAGs from the global oceans. (A) The geographic distribution of the cultivation-independent AOA MAGs reconstructed from global ocean microbiomes. The background world map was created using R, with land border line data downloaded from the Natural Earth website (<https://www.naturalearthdata.com>). (B) The phylogenetic tree of marine AOA, consisting of 281 high-quality MAGs (colored by black) and 11 pure species (colored by red). The tree was rooted by two terrestrial AOA species, *Nitrososphaera viennensis* EN76 and *N. gargensis* Ga9.2.

**Figure S2.** The correlations between environmental factors and their relationships with genome size of marine AOA. (A) The Pearson correlation between four environmental factors of 281 marine AOA MAGs. The distribution of marine AOA genome size along the gradients of salinity (B), water depth (C) and dissolved oxygen (D). Each point represents the selected genomes of marine ammonia-oxidizing archaea, which is colored by its genus. The relationships between archaeal genome size and environmental factors were fitted by a linear regression model, with the black line showing the relationship for all genomes, and the two-colored lines for two genera separately.

**Figure S3.** The relationship between water temperature and the predicted optimal growth temperature of AOA. The optimal growth temperature for marine AOA MAGs was predicted using tome, which is based on the proteome-wide amino acid composition (See the supplementary methods). The point represents the marine thaumarchaeotal genome size in each sample.

**Figure S4.** The relationships between marine AOA pangenomic features and their functional categories along the water temperature gradient. We performed pangenomic analyses for the selected genomes within each genus, then classified the orthologs into three groups, namely Total, Core, and Flexible (See the supplementary methods). The relationships between the three groups and temperature were fitted using the linear regression model, with the coefficient of determination (*R^2^*) in the significant model shown at the bottom left of each subpanel. (b) The heatmap of the correlations between water temperature and the archaeal ortholog counts. For each functional category, the correlation between the ortholog count and temperature was calculated using the Spearman coefficient. The asterisks indicate the significance of the correlation test, with * as *P* < 0.05, ** as *P* < 0.001, *** *P* < 0.0001.

**Figure S5.** The relationships between water temperature and the ortholog counts in the selected functional categories across two genera of marine AOA (A, B). The relationships were fitted using the linear regression model, with the coefficient of determination (*R^2^*) in the significant model shown at the bottom of each subpanel. The abbreviations for these functional categories were shown below, E: Amino acid transport and metabolism; G: Carbohydrate transport and metabolism; K: Transcription; L: Replication, recombination, repair; M: Cell wall/membrane/envelope biogenesis; O: Posttranslational modification, protein turnover, chaperones.

**Figure S6.** The comparison of the predicted optimal growth temperatures between the diverged clades and the remaining MAGs within two genera. The optimal growth temperature for marine AOA MAGs was predicted using tome, which is based on the proteome-wide amino acid composition (See the supplementary methods).

**Reference**

1.	Vernette C, Lecubin J, Sánchez P, Coordinators TO, Sunagawa S, Delmont TO, et al. The Ocean Gene Atlas v2.0: online exploration of the biogeography and phylogeny of plankton genes. *Nucleic Acids Res*. 2022;50:W516-W26.

2.	Eddy SR. Accelerated Profile HMM Searches. *PLOS Computational Biology*. 2011;7:e1002195.

3.	Nishimura Y, Yoshizawa S. The OceanDNA MAG catalog contains over 50,000 prokaryotic genomes originated from various marine environments. *Sci Data*. 2022;9:305.

4.	Parks DH, Imelfort M, Skennerton CT, Hugenholtz P, Tyson GW. CheckM: assessing the quality of microbial genomes recovered from isolates, single cells, and metagenomes. *Genome Research*. 2015;25:1043-55.

5.	Bayer B, Vojvoda J, Reinthaler T, Reyes C, Pinto M, Herndl GJ. *Nitrosopumilus adriaticus* sp. nov. and *Nitrosopumilus piranensis* sp. nov., two ammonia-oxidizing archaea from the Adriatic Sea and members of the class Nitrososphaeria. *International Journal of Systematic and Evolutionary Microbiology*. 2019;69:1892-902.

6.	Qin W, Heal KR, Ramdasi R, Kobelt JN, Martens-Habbena W, Bertagnolli AD, et al. *Nitrosopumilus maritimus* gen. nov., sp. nov., *Nitrosopumilus cobalaminigenes* sp. nov., *Nitrosopumilus oxyclinae* sp. nov., and *Nitrosopumilus ureiphilus* sp. nov., four marine ammonia-oxidizing archaea of the phylum Thaumarchaeota. *Int J Syst Evol Microbiol*. 2017;67:5067-79.

7.	Li M, Thieringer PH, Bayer B, Kellom M, Santoro AE. Genome sequence of *Nitrosopumilus* adriaticus CCS1 assembled from an ammonia-oxidizing enrichment culture. *Microbiology Resource Announcements*. 2024;13:e00692-24.

8.	Li H, Zhuang L, Cai H, Ni Y, Chu T, Chen L, et al. Nitrosarchaeum haohaiensis sp. Nov. CL1T: Isolation and Characterisation of a Novel Ammonia-Oxidising Archaeon From Aquatic Environments. *Environmental Microbiology Reports*. 2025;17:e70100.

9.	Dray Sep, Bauman D, Blanchet G, Borcard D, Clappe S, Gu{\'e}nard G, et al. adespatial: Multivariate Multiscale Spatial Analysis 2024 [

10.	Nayfach S, Shi ZJ, Seshadri R, Pollard KS, Kyrpides NC. New insights from uncultivated genomes of the global human gut microbiome. *Nature*. 2019;568:505-10.

11.	Richard HL, Peter FM, Ruth ZG. Introduction to bivariate and multivariate analysis. Glenview, IL: Scott, Foresman; 1980. 444 p.

12.	Groemping U. Relative Importance for Linear Regression in R: The Package relaimpo. *Journal of Statistical Software*. 2006;17:1 - 27.

13.	Emms DM, Kelly S. OrthoFinder: phylogenetic orthology inference for comparative genomics. *Genome Biology*. 2019;20:238.

14.	Buchfink B, Xie C, Huson DH. Fast and sensitive protein alignment using DIAMOND. *Nature Methods*. 2015;12:59-60.

15.	Li T, Yin Y. Critical assessment of pan-genomic analysis of metagenome-assembled genomes. *Briefings in Bioinformatics*. 2022;23.

16.	Hyatt D, Chen G-L, Locascio PF, Land ML, Larimer FW, Hauser LJ. Prodigal: prokaryotic gene recognition and translation initiation site identification. *BMC Bioinformatics*. 2010;11.

17.	Cantalapiedra CP, Hernández-Plaza A, Letunic I, Bork P, Huerta-Cepas J. eggNOG-mapper v2: Functional Annotation, Orthology Assignments, and Domain Prediction at the Metagenomic Scale. *Molecular Biology and Evolution*. 2021;38:5825-9.

18.	Huerta-Cepas J, Szklarczyk D, Heller D, Hernández-Plaza A, Forslund SK, Cook H, et al. eggNOG 5.0: a hierarchical, functionally and phylogenetically annotated orthology resource based on 5090 organisms and 2502 viruses. *Nucleic Acids Research*. 2018;47:D309-D14.

19.	Li G, Rabe KS, Nielsen J, Engqvist MKM. Machine learning applied to predicting microorganism growth temperatures and enzyme catalytic optima. *ACS Synthetic Biology*. 2019;8:1411-20.

20.	Jain C, Rodriguez-R LM, Phillippy AM, Konstantinidis KT, Aluru S. High throughput ANI analysis of 90K prokaryotic genomes reveals clear species boundaries. *Nature Communications*. 2018;9:5114.

21.	Ren M, Wang J. Phylogenetic divergence and adaptation of Nitrososphaeria across lake depths and freshwater ecosystems. *The ISME Journal*. 2022.

22.	Katoh K, Kuma K-i, Toh H, Miyata T. MAFFT version 5: improvement in accuracy of multiple sequence alignment. *Nucleic Acids Research*. 2005;33:511-8.

23.	Capella-Gutiérrez S, Silla-Martínez JM, Gabaldón T. trimAl: a tool for automated alignment trimming in large-scale phylogenetic analyses. *Bioinformatics*. 2009;25:1972-3.

24.	Price MN, Dehal PS, Arkin AP. FastTree 2 – approximately maximum-likelihood trees for large alignments. *PLOS ONE*. 2010;5:e9490.

25.	Minh BQ, Schmidt HA, Chernomor O, Schrempf D, Woodhams MD, von Haeseler A, et al. IQ-TREE 2: New Models and Efficient Methods for Phylogenetic Inference in the Genomic Era. *Molecular Biology and Evolution*. 2020;37:1530-4.

26.	Kalyaanamoorthy S, Minh BQ, Wong TKF, von Haeseler A, Jermiin LS. ModelFinder: fast model selection for accurate phylogenetic estimates. *Nature Methods*. 2017;14:587-9.

27.	Ren M, Hu A, Zhang L, Yao X, Zhao Z, Kimirei IA, et al. Acidic proteomes are linked to microbial alkaline preference in African lakes. *Water Research*. 2024;266:122393.

28.	Benjamini Y, Hochberg Y. Controlling the False Discovery Rate: A Practical and Powerful Approach to Multiple Testing. *Journal of the Royal Statistical Society: Series B (Methodological)*. 1995;57:289-300.
